# Role of luteinizing hormone elevation in outcomes of ovulation induction with letrozole for polycystic ovary syndrome

**DOI:** 10.3389/fmed.2023.1113840

**Published:** 2023-04-18

**Authors:** Wenyan Fu, Yanping Kuang

**Affiliations:** Department of Assisted Reproduction, Shanghai Ninth People’s Hospital, Shanghai Jiao Tong University School of Medicine, Shanghai, China

**Keywords:** LH elevation, ovulation induction, letrozole, polycystic ovary syndrome, oral contraceptives

## Abstract

**Introduction:**

The effect of elevated luteinizing hormone (LH) on the clinical outcomes of ovulation induction (OI) in infertile anovulatory patients with polycystic ovary syndrome (PCOS) remains controversial. This retrospective study included PCOS patients undergoing intrauterine insemination (IUI) following letrozole (LE) stimulation without OC pretreatment.

**Materials and methods:**

A retrospective cohort analysis was conducted in a single, academic ART center from January 2013 to May 2019. In total, 835 IUI cycles of PCOS patients treated with letrozole were collected for the analysis. Cohorts were separated based on the level of basal LH (bLH) and LH level after letrozole administration (LH_le_) during OI. OI response and reproductive outcomes were evaluated for each cohort.

**Results:**

No adverse effects of dysregulated levels of either bLH or LH_le_ on ovulation rate or reproductive outcomes were observed. Furthermore, the cohort of individuals with normal bLH and high LH_le_ levels, exclusive of LH surge, exhibited significantly higher rates of clinical pregnancy (30.3% vs. 17.3%, *p* = 0.002) and live birth (24.2% vs. 15.2%, *p* = 0.024) than those with normal bLH and normal LH_le._

**Conclusion:**

These results indicated that high LH levels in PCOS are not solid evidence of poor prognosis of letrozole-induced ovulation, while elevated LH_le_ may be a prospective predictor for better OI outcomes. It seems that preinhibition of LH secretion is not needed.

## Introduction

Polycystic ovary syndrome (PCOS), the most common endocrinopathy in women of reproductive age, is a major cause of female infertility worldwide (1, 2). As a multisystem disease, PCOS is characterized by hypothalamic–pituitary-ovarian axis dysfunction and metabolic disturbances, such as hyperandrogenaemia, hyperinsulinaemia/insulinaemia, elevated absolute levels of circulating luteinizing hormone (LH) and its relationship to follicle-stimulating hormone (FSH) levels and chronic anovulation (3, 4). A large percentage of patients fail to ovulate and conceive despite treatment with the first-line therapy of oral ovulation induction (OI) agents. Issues such as LH elevation during OI to achieve pregnancy have perplexed scientists (5). Up to 60% of PCOS patients are characterized by LH hypersecretion (6, 7). Some evidence suggests that excessive LH impedes oocyte maturity and fertilization (8) and eventually results in lower pregnancy and higher miscarriage rates (9). Other studies have reported that neither the quality of oocytes and embryos nor the rates of fertilization, implantation and pregnancy were impaired by increased endogenous LH levels (10, 11). In ovulation induction cycles stimulated with CC, no adverse effect of elevated endogenous LH or administration of exogenous LH on the probabilities of ovulation or achievement of pregnancy was observed (12). The clinical influence of LH increase needs to be further clarified (13).

Recently, based on its better efficiency of ovulation induction than CC (14), letrozole has been recommended as another first-line therapy. Abnormally high basal levels of LH and LH elevation after letrozole stimulation during OI are examined primarily in PCOS patients. To date, no clear data are available in the literature regarding the role of hypersecreted LH on the prognosis of ovulation induction with letrozole in PCOS patients. Meanwhile, OC pretreatment is commonly recommended to suppress LH secretion (15–17), attempting to refine the pregnancy outcomes in PCOS patients. Nevertheless, it remains controversial.

Hence, we performed this retrospective study to evaluate the effects of LH hypersecretion on the clinical outcomes of PCOS patients with letrozole-induced ovulation in intrauterine insemination (IUI) cycles, to provide a perspective on the prognostic role of elevated LH in infertile PCOS women who are scheduled for ovulation induction and to provide evidence regarding whether it is necessary to preabate LH secretion by OCs.

## Materials and methods

### Study population

A retrospective study was conducted by reviewing the medical records of infertile women with PCOS who attended the Department of Assisted Reproduction, Ninth People**’**s Hospital of Shanghai Jiao Tong University School of Medicine, for infertility treatment and underwent IUI therapy from January 2013 to May 2019. This study protocol was approved by the ethical committee of the hospital. PCOS was diagnosed according to the Rotterdam criteria of the European Society of Human Reproduction and Embryology (ESHRE) and American Society for Reproductive Medicine (ASRM) (13). All semen samples were evaluated according to the modified criteria of the World Health Organization (18). A total motile sperm count of at least 10 million, a processed total motile sperm (PTMS) count >2 million was one criterion for treatment with IUI (19, 20). All patients were diagnosed by hysterosalpingography, by hysteroscopy or underwent hysteroscopic hydrotubation of the oviduct and showed patency in at least one tube. Serum LH levels during ovulation induction were analy**z**ed to assess the relationship of LH with the outcome of IUI. As described previously (21), the cut-off level for basal LH was 10 ng/ml. Similarly, LH ≥ 10 ng/ml was defined as elevated LH following letrozole treatment.

### Ovulation induction protocol

Starting from the early follicular phase, commonly Days 2–4 of spontaneous menses.

or progesterone-induced withdrawal bleeding, the patients were treated with a dose of 5 mg letrozole (Jiangsu Hengrui Medicine Co., China) daily for 5 days. To assure the data comparability, we included only the patients treated with 5 mg letrozole per day for 5 days. Seven days after the first day that patients were administered letrozole, ultrasound monitoring and serum hormone analysis were performed. If there was no dominant follicle or the leading follicle was <14 mm, 75 IU human menopausal gonadotropin (HMG, Anhui Fengyuan Pharmaceutical Co.) every other day was prescribed. Thereafter, the endometrium, follicular development and sex hormone levels were monitored as necessary. If needed, the administered dose of HMG was increased by 37.5 IU incrementally, while the frequency of HMG use was increased up to daily. Moreover, a low dose of oral estrogen was added to improve the endometrium (EM) as needed. When at least one follicle reached a mean diameter of 17–18 mm, the EM thickness reached 7 mm, and ideally, when E2 levels were > 150 pg./ml, ovulation was triggered with human chorionic gonadotropin (hCG; 5,000 IU; Lizhu Pharmaceutical Trading Co.). IUI was then performed at the appropriate time.

### Outcome

Serum β-human chorionic gonadotropin (β-hCG) was assessed 17 days after IUI. A biochemical pregnancy was defined by a β-hCG concentration > 10 mIU/ml. Patients with a positive β-hCG test underwent an ultrasound test 2 weeks later. If at least one gestational sac was observed, it was diagnosed as a clinical pregnancy. A live birth was defined as a live-born baby after 24 or more gestational weeks. A pregnancy was defined as clinical pregnancy loss if it was eventuated in a spontaneous or therapeutic abortion that occurred after clinical pregnancy. Since many patients who underwent two failed IUI cycles preferred treatment with *in vitro* fertilization and embryo transfer (IVF-ET), all cumulative rates were calculated after two cycles.

### Statistical analysis

The distributions of categorical variables were compared with the chi-square test, while continuous variables were compared with one-way ANOVA. Continuous variables are summarized as the mean ± standard deviation. Multivariate analysis and 1:1 PS matching were used to adjust the effect of baseline characteristics and treatment arms. All analyses were performed with SPSS software (SPSS Inc., Version 22.0, Chicago, IL, USA). A *p-*value <0.05 was considered statistically significant.

## Results

### Baseline characteristics, OI response and reproductive outcomes of PCOS patients with dysregulation of endogenous LH

To analyze the effect of aberrant LH levels on the reproductive outcome of PCOS patients with ovulation induction, 835 cycles fulfilled the inclusion criteria and were divided into 3 groups: patients with normal levels of both basal LH and LH following treatment with letrozole for 5 days (N-bLH-N-LH_le_), patients with normal basal LH but elevated LH after letrozole administration (N-bLH-H-LH_le_), and others expressing high levels of bLH (H-bLH). Apart from similar infertility durations and infertility types, the patients with normal endogenous LH were older and characterized by a higher BMI than the other patients (Table 1). PCOS is a syndrome with high heterogeneity. We intended to define the PCOS subpopulation numbers according to the NIH subclassification (22). Since most testosterone assays have poor sensitivity and accuracy in the female ranges (23) and this was a retrospective analysis, we did not have sufficient testosterone level data and could not accurately classify the phenotypes in this study. Thus, we characterized patients separately based on the number of antral follicles, ovulatory function, and hyperandrogenism and found no significant difference between the groups. Moreover, male fertile history, sperm quality and processed total motile sperm count were evaluated, and comparable characteristics were observed between the three groups.

**Table 1:** Baseline characteristics, ovulation induction response.

Characteristics	N-bLH-N-LH_le_^1^	N-bLH-H-LH_le_^2^	H-bLH^3^	*p*-value
Total (%)	533 (63.8)	231 (27.7)	71 (8.5)	
Age (female, years)	34.507 ± 0.167	33.675 ± 0.231	33.423 ± 0.450	0.004
Infertility duration (years)	3.184 ± 0.099	3.043 ± 0.150	3.451 ± 0.272	0.407
**Infertility type**				
Primary	384 (72.0)	178 (77.1)	59 (83.1)	0.073
Secondary	149 (28.0)	53 (22.9)	12 (16.9)	
BMI	24.482 ± 0.182	23.031 ± 0.269	22.473 ± 0.404	0.000
Ovulatory function (%)				0.469
Normal function	9 (1.7)	2 (0.9)	2 (2.8)	
Dysfunction	524 (98.3)	229 (99.1)	69 (97.2)	
AFC				0.372
<12	48 (9.0)	15 (6.5)	4 (5.6)	
≥12	485 (91.0)	216 (93.5)	67 (94.4)	
Male fertile history, sperm quality				0.786
Primary sterility, normozoospermia	290 (54.4)	134 (58.0)	45 (63.4)	
Primary sterility, mild oligo/asthenozoospermia	86 (16.1)	32 (13.9)	11 (15.5)	
Secondary sterility, normozoospermia	135 (25.3)	56 (24.2)	13 (18.3)	
Secondary sterility, mild oligo/asthenozoospermia	22 (4.1)	9 (3.9)	2 (2.8)	
PTMS (Million)				0.711
5≤	105 (19.7)	42 (18.2)	18 (25.4)	
5<, ≥10	201 (37.7)	100 (43.3)	26 (36.6)	
10<, ≥15	142 (26.6)	54 (23.4)	17 (23.9)	
>15	85 (15.9)	35 (15.2)	10 (14.1)	
Clinical/ biochemical hyperandrogenism				0.910
No	122 (23.2)	54 (23.6)	17 (23.9)	
Yes	12 (2.3)	3 (1.3)	2 (2.8)	
Unknown	391 (74.5)	172 (75.1)	52 (73.2)	
**Stimulation protocol**				
LE	85 (15.9)	8 (3.5)	10 (14.1)	0.000
LE + HMG	448 (84.1)	223 (96.5)	61 (85.9)	
Does of HMG	421.875 ± 16.704	508.857 ± 25.318	404.508 ± 35.208	0.007
Days of HMG administration	3.505 ± 0.114	3.928 ± 0.159	3.197 ± 0.275	0.035
E2 level at the day of trigger	201.618 ± 6.067	297.659 ± 18.971	295.656 ± 27.067	0.000
FC > 14 mm	1.556 ± 0.052	2.022 ± 0.114	1.967 ± 0.134	0.000
EM	10.042 ± 0.109	10.093 ± 0.153	9.444 ± 0.274	0.127
Ovulation (%)	529 (99.2)	229 (99.1)	71 (100)	0.744

bLH, basal luteinizing hormone; LE, letrozole; LH_le_, LH level after LE treatment; BMI, body mass index; HMG, human menopausal gonadotropin; FC, follicle; EM, endometrium; AFC, antral follicles; PTMS, processed total motile sperm.^1^bLH < 10mIU/mL and LH_le_ < 10mIU/mL; ^2^bLH < 10mIU/mL and LH_le_ ≥ 10mIU/mL; ^3^bLH ≥ 10mIU/mL. Data are presented as mean ± SD for continuous variables and *n* (%) for dichotomous variables. All *p*-values were assessed with the use of χ^2^ or Student *t-*test.

**Figure 1:**
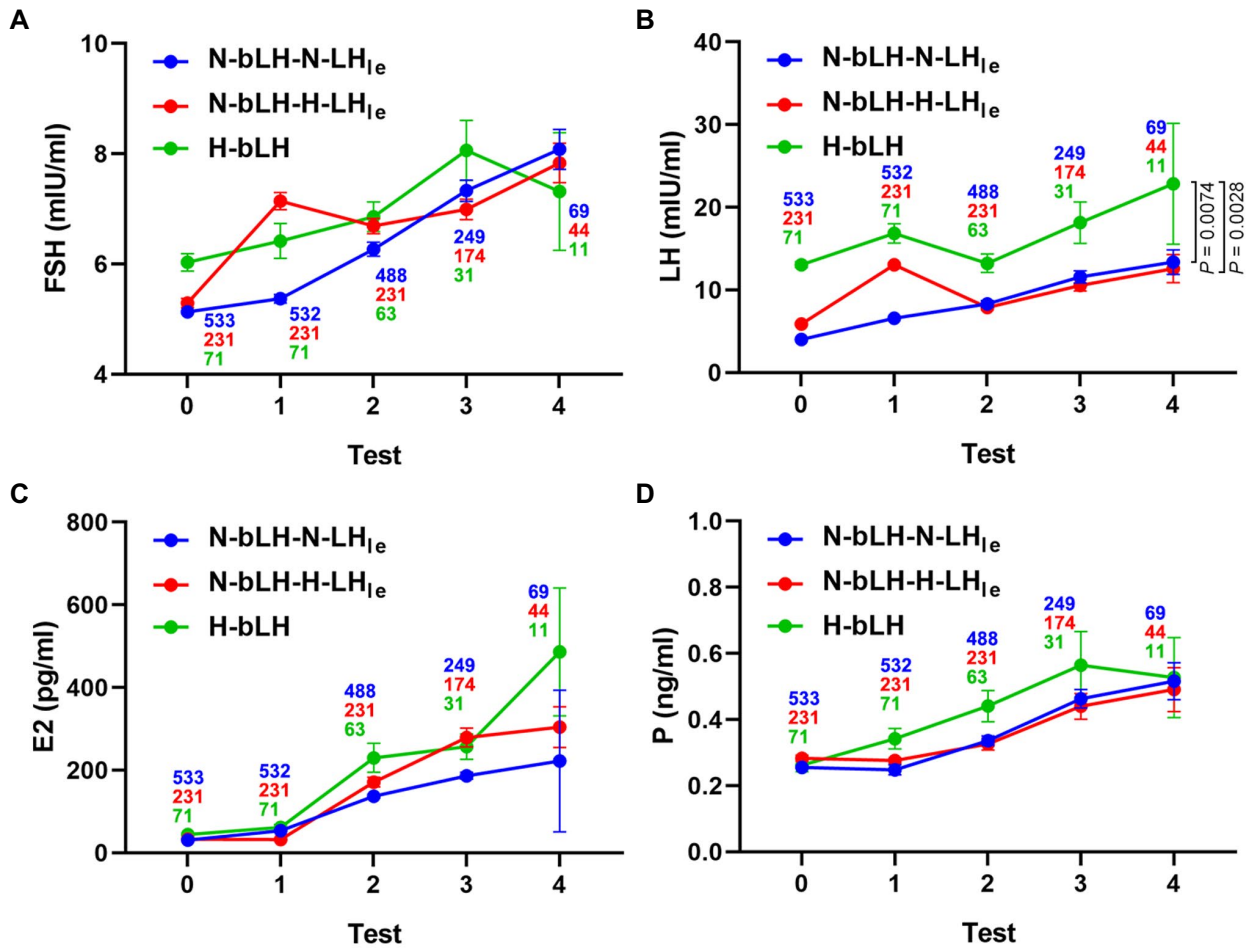
Hormone profile of different LH groups during ovulation induction with letrozole. **(A)** Serum FSH level. **(B)** Serum LH level. **(C)** Serum E2 level. **(D)** Serum P level. Test 0 indicates the basal hormone levels. Test 1 refers to the hormone levels following LE treatment for 5 days. In tests 2, 3, and 4, hormone levels were measured several times until the trigger day.

Additionally, the hormone profile during OI is depicted in Figure 1. There were no considerable differences in the change patterns for FSH, E2, and P4, but a difference in the change pattern for LH was observed among these groups. The E2 level remained low during treatment with letrozole and increased gradually with the growth of follicles in all groups. The circulating LH level of patients with high bLH was comparatively higher than that of patients with normal bLH throughout the entire OI process. Especially in the N-bLH-H-LH_le_ and H-bLH groups, the serum LH increased and peaked on the last day of letrozole treatment, decreased at the early days after stopping the use of letrozole, and then increased slightly and remained at a low level until the occurrence of the LH surge. Nevertheless, the P4 level remained low throughout ovarian stimulation.

To investigate the role of elevated LH in the prognosis of OI, we further explored the OI response and outcomes of different groups. Compared with the other two groups, the N-bLH-H-LH_le_ group comprised a greater percentage of patients requiring HMG for ovarian stimulation (96.5%, *p* = 0.000, Table 1). Therefore, the OI response was analyzed in these patients, excluding those treated with a single letrozole. Consistent with the highest percentage of HMG, the N-bLH-H-LH_le_ cohort showed a much higher total dose of HMG (*p* = 0.007) and a longer duration of HMG administration (*p* = 0.035) than the other groups. As shown in Table 1, patients with N-bLH-N-LH_le_ developed fewer follicles larger than 14 mm and concordantly lower E2 levels on the trigger day. However, neither the EM thickness on the trigger day nor the overall ovulation rates were significantly different between the three groups. These results suggested that dysregulation of LH in PCOS patients had no adverse effect on follicle maturation and ovulation and that combined usage of HMG with letrozole reversed the poor ovarian response, which may be correlated with the abnormally elevated LH followed by letrozole treatment.

Taking the N-bLH-N-LH_le_ group as the control, the N-bLH-H-LH_le_ group exhibited remarkably higher rates of biochemical pregnancy (31.2% vs. 17.8%, *p* = 0.002), clinical pregnancy (30.3% vs. 17.3%, *p* = 0.002), and live birth (24.2% vs. 15.2%, *p* = 0.024), while the H-bLH group did not show obvious differences in the rates of either pregnancy or live birth. No significant difference in the clinical pregnancy rate was detected among the three groups. Furthermore, cumulative rates were calculated based on two IUI cycles. Likewise, compared with the control, the N-bLH-H-LH_le_ cohort had significantly higher cumulative rates of biochemical pregnancy (39.3% vs. 22.7%, *p* = 0.005), clinical pregnancy (38.1% vs. 22.0%, *p* = 0.006), and live birth (30.2% vs. 19.1%, *p* = 0.039). Similar cumulative rates of pregnancy and live birth were observed between the H-bLH and N-bLH-N-LH_le_ groups (Table 2). To further exclude the functions played by ageing and obesity, data were adjusted by 1:1 PS matching. Consistently, taking the N-bLH-N-LH_le_ group as a control, the N-bLH-H-LH_le_ and H-bLH groups exhibited identical reproductive rates (Table 3).

**Table 2:** Live birth, pregnancy, and pregnancy loss*.

Outcome		*N* (%)	Univariate		Multivariate	
			Rate ratio (95% CI)	*P-*value	Rate ratio (95% CI)	*P-*value
Biochemical pregnancy^a^						
	^1^N-bLH-N-LH_le_	95 (17.8)	ref		ref	
	^2^N-bLH-H-LH_le_	72 (31.2)	2.088 (1.462–2.980)	0.000	1.850 (1.261–2.714)	0.002
	^3^H-bLH	15 (21.1)	1.235 (0.670–2.276)	0.499	1.132 (0.604–2.122)	0.699
Clinical pregnancy^b^						
	N-bLH-N-LH_le_	92 (17.3)	ref		ref	
	N-bLH-H-LH_le_	70 (30.3)	2.084 (1.455–2.986)	0.000	1.847 (1.254–2.722)	0.002
	H-bLH	14 (19.7)	1.177 (0.629–2.202)	0.609	1.076 (0.565–2.048)	0.824
Live birth^c^						
	N-bLH-N-LH_le_	81 (15.2)	ref		ref	
	N-bLH-H-LH_le_	56 (24.2)	1.786 (1.218–2.618)	0.003	1.610 (1.065–2.434)	0.024
	H-bLH	10 (14.1)	0.915 (0.450–1.859)	0.806	0.828 (0.401–1.710)	0.610
Clinical pregnancy loss^d^						
	N-bLH-N-LH_le_	11 (12.0)	ref		ref	
	N-bLH-H-LH_le_	14 (20.0)	1.841 (0.779–4.350)	0.164	1.612 (0.589–4.414)	0.353
	H-bLH	4 (28.6)	2.945 (0.787–11.021)	0.109	3.390 (0.851–13.508)	0.083
Cumulative biochemical pregnancy rate						
	N-bLH-N-LH_le_	91 (22.7)	ref		ref	
	N-bLH-H-LH_le_	68 (39.3)	1.799 (1.324–2.444)	0.000	1.610 (1.154–2.247)	0.005
	H-bLH	15 (34.6)	1.644 (0.949–2.849)	0.076	1.514 (0.865–2.650)	0.146
Cumulative clinical pregnancy rate						
	N-bLH-N-LH_le_	88 (22.0)	ref		ref	
	N-bLH-H-LH_le_	66 (38.1)	1.806 (1.323–2.464)	0.000	1.616 (1.151–2.267)	0.006
	H-bLH	14 (33.5)	1.586 (0.899–2.797)	0.111	1.456 (0.818–2.594)	0.202
Cumulative live birth rate						
	N-bLH-N-LH_le_	78 (19.1)	ref		ref	
	N-bLH-H-LH_le_	52 (30.2)	1.638 (1.165–2.303)	0.005	1.478 (1.020–2.141)	0.039
	H-bLH	10 (21.4)	1.253 (0.647–2.430)	0.503	1.137 (0.580–2.227)	0.708

^*^Cumulative rates were calculated based on two IUI cycles. bLH, basal luteinizing hormone; LE, letrozole; LH_le_, LH level after LE treatment; ^1^bLH < 10 mIU/mL and LH_le_ < 10 mIU/mL; ^2^bLH < 10 mIU/mL and LH_le_ ≥ 10 mIU/mL; ^3^bLH ≥ 10 mIU/mL. Age, BMI, Stimulation protocol, Does of HMG, Days of HMG administration, E2 level at the day of trigger, FC > 14 mm were used for multivariate analysis. ^a^Biochemical pregnancy was defined by a serum hCG of > 10 mIU/ml. ^b^Clinical pregnancy was defined by observation of intrauterine gestational sac on ultrasonography. ^c^Live birth was defined as delivery of any viable infant≧28 weeks gestation. ^d^Clinical pregnancy loss was defined as conceptions that did not result in live birth. All *P-*values were assessed with Logistic regressive analysis.

**Table 3:** Live birth, pregnancy, and pregnancy loss adjusted by 1:1 PS matching^*^.

	^1^N-bLH-N-LH_le_ vs. ^2^N-bLH-H-LH_le_			N-bLH-N-LH_le_ vs. ^3^H-bLH			N-bLH-H-LH_le_ vs. H-bLH		
Outcome	^e^N (%)	RR (95% CI)	P	^f^N (%)	RR (95% CI)	P	^g^N (%)	RR (95% CI)	P
Biochemical pregnancy^a^	32 (15.8) vs. 59 (29.2)	2.192 (1.350–3.558)	0.001	12 (19.7) vs. 14 (23.0)	1.216 (0.510–2.899)	0.659	16 (27.6) vs. 13 (22.4)	0.758 (0.326–1.764)	0.521
Clinical pregnancy^b^	29 (14.4) vs. 57 (28.2)	2.345 (1.424–3.861)	0.001	12 (19.7) vs. 13 (21.3)	1.106 (0.459–2.666)	0.823	16 (27.6) vs. 12 (20.7)	0.685 (0.291–1.614)	0.387
Live birth^c^	27 (13.4) vs. 47 (23.3)	1.965 (1.168–3.307)	0.011	12 (19.7) vs. 9 (14.8)	0.707 (0.274–1.824)	0.473	12 (20.7) vs. 8 (13.8)	0.613 (0.230–1.634)	0.328

^*^Cumulative rates were calculated based on two IUI cycles. bLH, basal luteinizing hormone; LE, letrozole; LH_le_, LH level after LE treatment; RR, rate ratio; ^1^bLH < 10 mIU/mL and LH_le_ < 10 mIU/mL; ^2^bLH < 10 mIU/mL and LH_le_ ≥ 10 mIU/mL; ^3^bLH ≥ 10 mIU/mL. ^a^Biochemical pregnancy was defined by a serum hCG of > 10 mIU/ml. ^b^Clinical pregnancy was defined by observation of intrauterine gestational sac on ultrasonography. ^c^Live birth was defined as delivery of any viable infant ≧ 28 weeks gestation. All *p*-values were assessed with Logistic regressive analysis. ^e^Two hundred and thirty cycles in each group; ^f^Sixty-one cycles in each group; ^g^Fifty-eight cycles in each group.

## Discussion

Our study collected and analyzed the data of PCOS patients who underwent IUI with letrozole stimulation but not OC pretreatment. The administered dose of letrozole differed based on the length of the menstrual cycle. To improve comparability, we included only patients treated with 5 mg letrozole per day for 5 days and then investigated the effect of high LH levels on the outcome of letrozole-induced ovulation. Here, the low E2 level, small follicle size and unchanged P level suggested that the increased LH_le_ was not an LH surge. In the present data, the N-bLH-N-LH_le_ group developed the lowest number of follicles >14 mm and, subsequently, the lowest level of E2 at the trigger day, which may be due to this group having the oldest age, highest BMI, and lower HMG dosage. Furthermore, the patients characterized by N-bLH-H-LH_le_ showed the highest percentage of LE + HMG protocol usage as well as the highest dose and the longest duration of HMG for ovarian stimulation, suggesting unique reproductive endocrinal characteristics of the cohort, such as the LH_le_ level increase. As a result, neither a high level of bLH nor elevated LH following letrozole administration had an adverse effect on the clinical outcome of ovulation induction with letrozole. Surprisingly, compared with the other two groups, the N-bLH-H-LH_le_ group displayed better reproductive outcomes, which illustrated that, despite a poorer ovarian response, an elevated LH_le_ may be a promising predictor of better clinical outcome following ovarian induction with letrozole. Nevertheless, additional investigation is needed to elucidate the mechanism.

Several days after letrozole administration, ultrasound monitoring and serum hormone analysis were performed. If there was no dominant follicle or the leading follicle was <14 mm, low dose HMG was prescribed. The combination of letrozole and gonadotropin in OI cycle results in shorter OI duration and less cycle cancellation rate for poor ovarian response (24, 25). In our study, the average dose of HMG administration per cycle was about 400-500 IU. The average cost of HMG was about only $16.65-$20.4 ($3.05 per 75 U HMG). Ovulation induction by adding HMG to letrozole is low-cost and time-saving, and may be more cost-effective than letrozole alone.

Oral contraceptives have long been considered an option to treat anovulatory infertility in PCOS by reducing LH and androgen plasma levels, restoring normal and adequate spontaneous episodic gonadotropin discharge, decreasing ovarian volumes and regularizing menstrual cycles for planning ovulation induction programs (26, 27). Branigan EF et al. showed that OI with CC following OC pretreatment yielded higher pregnancy rates for PCOS women resistant to CC (28). In contrast, no significantly better outcome following preintervention with OCs was observed in another clinical trial (29). Recently, OCs, combined with GnRH agonists or antagonists, have been used in IVF programs to prevent a premature LH peak (30–32) and improve reproductive outcomes in PCOS patients (33, 34). However, growing concerns have been raised regarding the potential detrimental efficiency of OCs usage on reproductive outcomes for patients undergoing infertility treatment (35–38). In the 2019 ESHRE guideline of ovarian stimulation for IVF/ICSI, OCs pretreatment is not recommended in the GnRH antagonist protocol because of its reduced efficacy (39). OCs usage for 3–6 months was previously suggested to improve the ovarian response to CC and resolve CC resistance (28). The effect of OCs pretreatment on the ovarian response and reproductive outcomes of letrozole-induced ovulation is unclear. In our study, the ovulation rates following letrozole stimulation with HMG reached 99–100% for all patients and showed no significant difference among groups. Pretreatment with OCs to improve the ovarian response to letrozole seems unnecessary. Notably, ovarian costimulation with letrozole and HMG without preintervention is more affordable and time-saving than 3–6 months of OCs treatment before letrozole stimulation. Given the continuously increased E2 and maintained low P levels throughout the process of ovulation induction, neither bLH nor LH_le_ increase impaired the quality and maturation of follicles. Together with the pregnancy outcomes, all the results observed in our study implied that systematic pretreatment with OCs may not be needed for PCOS patients before ovulation induction with letrozole.

Our study also has limitations. First, there were a limited number of patients in the group with high bLH. Therefore, the samples may not fully recapitulate the population. Additionally, the present study is a retrospective study comprising only cases without OCs pretreatment. Prospective studies are needed to include more cases according to NIH subclassification and discuss the necessity of OCs pretreatment for ovulation induction in PCOS patients.

In summary, the endogenous LH increase of PCOS patients has no correlation with reproductive outcomes of ovulation induction with letrozole. Moreover, an elevated LH_le_ level, which should be distinguished from an LH surge and be carefully inhibited with, may be a positive reaction to ovulation induction and predict a better reproductive prognosis for PCOS patients.

## Conclusion

In conclusion, this study revealed that LH elevation in PCOS patients are not accurate predictors of poor prognosis of OI mediated by letrozole. There was no need to preinterfer with the LH increase before OI.

## Data availability statement

The raw data supporting the conclusions of this article will be made available by the authors, without undue reservation.

## Ethics statement

The studies involving human participants were reviewed and approved by the ethical committee of Shanghai Ninth People’s Hospital. The patients/participants provided their written informed consent to participate in this study.

## Author contributions

WF and YK designed and performed the research, analyzed the data, and wrote the manuscript. All authors contributed to the article and approved the submitted version.

## Funding

This study was supported by the National Natural Science Foundation of China (grant nos. 81771538, 82271732 and 81903140), the General Program of National Natural Science Foundation (grant no. 2018YFC10030), the Shanghai Medical Innovation Research Special Project (22Y21900800), the Shanghai Rising-Star Program (23QA1405800) and the Shanghai Sailing Program (19YF1438600).

## Conflict of interest

The authors declare that the research was conducted in the absence of any commercial or financial relationships that could be construed as a potential conflict of interest.

## Publisher’s note

All claims expressed in this article are solely those of the authors and do not necessarily represent those of their affiliated organizations, or those of the publisher, the editors and the reviewers. Any product that may be evaluated in this article, or claim that may be made by its manufacturer, is not guaranteed or endorsed by the publisher.
